# Influence of ordered L1_2_ precipitation on strain-rate dependent mechanical behavior in a eutectic high entropy alloy

**DOI:** 10.1038/s41598-019-42870-y

**Published:** 2019-04-23

**Authors:** Bharat Gwalani, Sindhura Gangireddy, Yufeng Zheng, Vishal Soni, Rajiv S. Mishra, Rajarshi Banerjee

**Affiliations:** 1 Advanced Materials and Manufacturing Processes Institute, University of North Texas, Denton, TX 76207 USA; 2 Materials Science and Engineering, University of North Texas, Denton, TX 76207 USA; 3 Department of Materials Science and Engineering, The Ohio State University, Columbus, OH 4310 USA; 4 Present Address: Physical and Computational Sciences Directorate, Pacific Northwest National Laboratory, Richland, USA

**Keywords:** Metals and alloys, Mechanical properties

## Abstract

Recent studies indicate that eutectic high-entropy alloys can simultaneously possess high strength and high ductility, which have potential industrial applications. The present study focuses on Al_0.7_CoCrFeNi, a lamellar dual-phase (*fcc* + B2) precipitation-strengthenable eutectic high entropy alloy. This alloy exhibits an *fcc* + B2 (B2 with *bcc* nano-precipitates) microstructure resulting in a combination of the soft and ductile *fcc* phase together with hard B2 phase. Low temperature annealing leads to the precipitation of ordered L1_2_ intermetallic precipitates within the *fcc* resulting in enhanced strength. The strengthening contribution due to fine scale L1_2_ is modeled using Orowan dislocation bowing and by-pass mechanism. The alloy was tested under quasi-static (strain-rate = 10^−3^ s^−1^) tensile loading and dynamic (strain-rate = 10^3^ s^−1^) compressive loading. Due to the fine lamellar microstructure with a large number of *fcc*-*bcc* interfaces, the alloy show relatively high flow-stresses, ~1400 MPa under quasi-static loading and in excess of 1800 MPa under dynamic loading. Interestingly, the coherent nano-scale L1_2_ precipitate caused a significant rise in the yield strength, without affecting the strain rate sensitivity (SRS) significantly. These lamellar structures had higher work hardening due to their capability for easily storing higher dislocation densities. The back-stresses from the coherent L1_2_ precipitate were insufficient to cause improvement in twin nucleation, owing to elevated twinning stress under quasi-static testing. However, under dynamic testing high density of twins were observed.

## Introduction

While high entropy alloys (HEAs), or multi-principal-component alloys^1–5^ have attracted the attention of the worldwide scientific community in recent years, most of the focus has been on discovering and investigating single phase solid solution alloys, presumably stabilized by their configuration entropy of mixing, especially at high temperatures. Typically, HEAs based on the face-centered cubic (*fcc*) crystal structure, such as the widely investigated Al_0.1_CoCrFeNi and CoCrFeMnNi alloys^1–5^, have been reported to exhibit excellent room temperature tensile ductility but rather poor strengths, whereas the body-centered cubic (*bcc*) based HEAs, like those containing refractory elements based on Nb-Mo-Ta-W-V-Mo etc^6–8^, exhibit very high compressive strength values but very limited ductility under tensile loading. Additionally, two-phase *bcc* +B2 HEAs, such as FeNiMnAl^9^, exhibiting a fine scale spinodal-like microstructure, exhibit phenomenal compressive yield strengths (~2350 MPa), but no tensile ductility.

A commonly adopted strategy to overcome this strength-ductility tradeoff is to develop multi-phase microstructures, involving at least one ductile/soft and one hard/strong phase, mixed in the appropriate proportion. Common examples include duplex alloys, such as 2205 stainless steel, which have a hard *bcc* phase to increase the strength of the alloy and a soft *fcc* phase to maintain the tensile ductility while deformation. The goal has been on fine-tuning the alloy composition and/or processing conditions in order to obtain a material with a good balance between ductility and strength. Eutectic alloys, often exhibiting a two-phase lamellar microstructure, offer such a possibility, and have been investigated for many years in different classes of alloys. Eutectic morphologies are characterized by the simultaneous growth of two (or more) phases from the liquid. A recently developed new class of eutectic HEAs (EHEAs), have attracted a lot of attention due to their promising mechanical properties^10–18^. These eutectic HEAs typically exhibit a lamellar *fcc* + B2 microstructure, with the ability to tune the composition of both phases over a wide range due to their inherent complexity. The most extensively investigated EHEAs include Fe_30_Ni_20_Mn_35_Al_15_^10–12^ and AlCoCrFeNi_2.1_^11–18^. Other EHEA compositions reported are CoCrFeNiMnPd_x_^19^ and CoCrFeNiZr_x_^20^. Most reports in the published literature on such alloys are based on the as-cast microstructure without any subsequent thermomechanical treatments.

The present study focuses on the marginally hypo-eutectic HEA with the nominal composition of Al_0.7_CoCrFeNi (15 at. % Al and 21.25 at.% each of Co, Cr, Fe, Ni). While a few previous investigations on this HEA have reported a lamellar two-phase *fcc*/B2microstructure in this alloy^21^, a detailed investigation of the microstructure of this alloy and its evolution during post-solidification thermo-mechanical processing forms the basis of the present study. Furthermore, the present study also investigates the influence of such heat-treatments on the mechanical behavior of this alloy under both quasi-static low strain rate tensile loading conditions, as well as dynamic strain rate compression conditions. The results clearly indicate that with suitable heat treatments the *fcc* phase in the lamellar two-phase microstructure is further strengthened by forming ordered L1_2_ nano precipitates, without compromising the tensile ductility. The *fcc* + L1_2_/*bcc* + B2 microstructure in this alloy exhibited a tensile yield strength close to 1000 MPa (~990 MPa), ultimate tensile strength (UTS) ~1400 MPa and elongation to failure of ~13% under quasi-static loading (strain rate of 10^−3^) whereas flow stresses reaching to over 1800 MPa without failure under dynamic loading (strain rate of 2 × 10^3^). Microstructural assessment after deformation revealed substantial deformation twinning in this alloy at higher strain rates.

## Experimental Methods

### Materials processing

**Figure 1:**
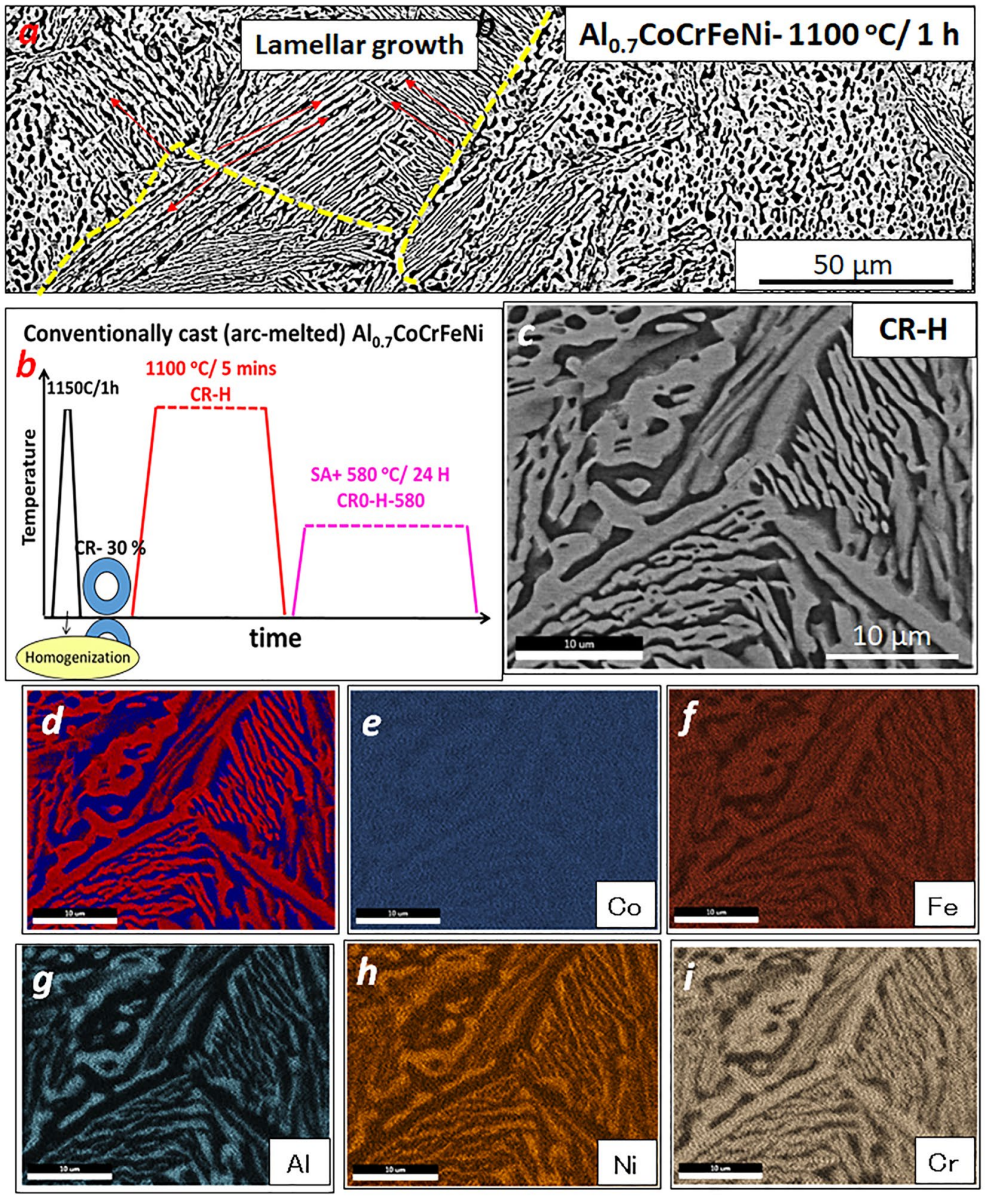
(**a**) Low magnification SEM BSED image from the Al_0.7_CoCrFeNi **CR-H** condition (**b**) Al_0.7_CoCrFeNi Processing Route (**c**–**i**) SEM-EDS characterization of the Al_0.7_CoCrFeNi **CR-H** condition.

The alloy of composition Al_0.7_CoCrFeNi (15 at. % Al and 21.25 at.% each of Co, Cr, Fe, Ni) was produced by melting elemental Al, Co, Cr, Fe and Ni mixed in appropriate proportions. Figure 1(b) charts the processing route followed to obtain different microstructures and resulting properties. The as cast alloy was homogenized at 1150 °C for 1 h to annihilate the dislocations and reduce micro-segregations from casting process to assist in cold deformation of the alloy. The SEM microstructure from this condition is shown in Fig. 1(a). The backscattered electron diffraction (BSED) image in Fig. 1(a) shows a two phase eutectic type of microstructure with a bright contrast phase and dark contrast phase organized in lamellar arrangement. The grain boundaries are highlighted by the yellow lines and lamellae directions are shown using red arrows in the figure. The alloy was then rolled at room temperature to 30% reduction in thickness. A subsequent annealing at 1100 ^o^C for 5 mins was done to homogenize and recrystallize the alloy at high temperature. The cold-rolled and homogenized condition is referred as ***CR-H*** hereafter. Another low temperature annealing treatment was done at 580 ^o^C for 24 h on the CR-H condition, this is referred as the ***CR-H-580*** condition.

### Microstructural characterization

To analyze the microstructure of the material after processing, pieces were cut and metallographically polished in stages. Final mirror surface finish was achieved by 0.02 µm colloidal silica gel VibroMet^TM^ polishing. The crystal orientations and elemental composition were characterized by electron backscattered diffraction (EBSD) and energy dispersive spectroscopy (EDX), respectively, in a FEI Nova Nano scanning electron microscope (SEM). Lift-out samples for transmission electron microscopy (TEM) and atom probe tomography (APT) were prepared using an FEI Nova 200 dual beam focused ion beam (FIB). TEM analysis was conducted using an FEI Tecnai F20 operated at 200 kV fitted with a STEM-EDS detector. To measure the composition, energy dispersive spectroscopy (Super-X system) equipped on an FEI-TITAN G2 TEM microscope was used in the HAADF STEM mode operating at 300 kV and the results were analyzed with FEI’s ES vision software version 6. The APT experiment was performed with a local electrode atomprobe (LEAP 3000X) with a target evaporation rate of 0.5% per pulse at 45 K. APT data reconstruction and quantitative analysis were performed using a CAMECA visualization and analysis software (IVAS) 3.6.8.

### Mechanical characterization

Dog-bone-shaped tensile specimens with gauge length 3 mm were machined from the rolled and annealed sheets by Electrical Discharge Machining (EDM). Both sides of the specimen were ground using SiC paper to achieve final thickness of ~0.7 mm and gauge width of ~1.0 mm. Tensile tests were performed at an engineering strain rate of 10^−3^ s^−1^. Each test was performed at least three times to ensure consistency; representative results are reported herein. These quasi-static tests used a LVDT (linear variable displacement transformer) extensometer to perform at least four independent tests to obtain tensile elongation and strength data. Yield strength, ultimate tensile strength, and elongation to failure were determined from the uniaxial tensile stress–strain curves. Cubes of 3 mm were compressed dynamically using split Hopkinson pressure bar (SHPB) testing machine at 2 × 10^3^ s^−1^ strain rate.

## Results and Discussion

### Influence of thermo-mechanical processing on microstructure

The SEM microstructure from the CR-H condition of the Al_0.7_CoCrFeNi HEA is shown in Fig. 1. The SEM backscatter images in Fig. 1(a,c) reveal the lamellar morphology of the two-phase eutectic in this alloy. Depending on the orientation of these lamellar eutectic colonies, with respect to a two-dimensional plane of sectioning, the lamellar structure could appear more globular-like, as seen in some regions of Fig. 1(a). The partitioning of elements into these phases, was determined using SEM-EDS. The dark contrast phase highlighted in blue color in Fig. 1(d), is rich in Al and Ni whereas Cr, Fe and Co partitions to the bright contrast phase(red). The SEM-EDS elemental maps of all the elements are shown in Fig. 1(e–i).

**Figure 2:**
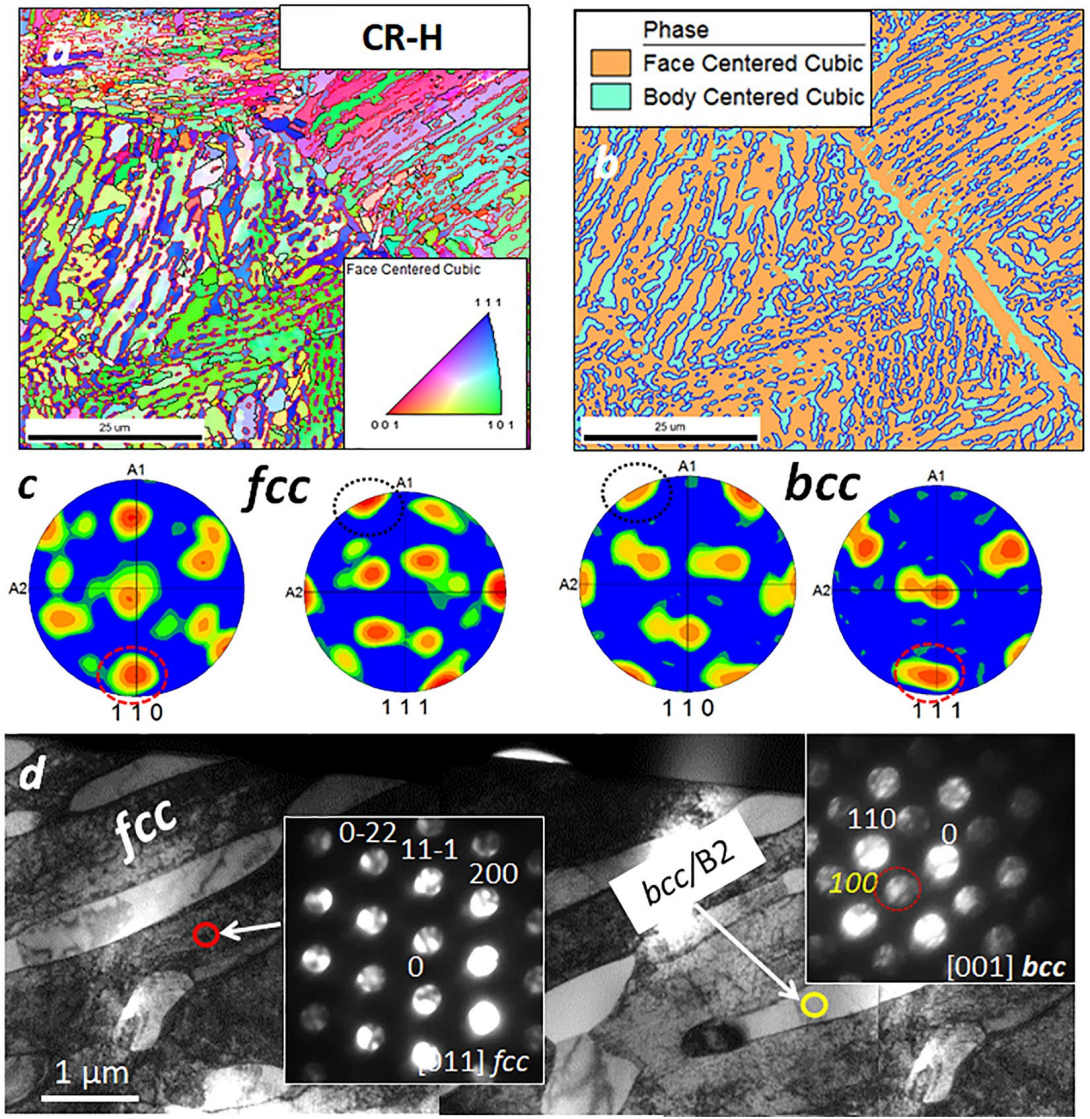
(**a**,**b**) SEM-EBSD characterization of the Al_0.7_CoCrFeNi **CR-H** condition. (**c**) EBSD pole figures from the *fcc* and *bcc* regions showing the orientation relationship. (**d**) BFTEM from **CR-H** condition with SAD from the two phase (shown in inset).

EBSD and TEM experiments were conducted to understand the crystallography of each phase. Figure 2(a–c) show the EBSD results and Fig. 2(d) shows a montage of bright field TEM images from the CR-H condition. The inverse pole figure (IPF) (inset in Fig. 2(a)) and the phase map (Fig. 2(b)) were obtained by consistently indexing the two phases as *fcc* and *bcc*. Figure 2(b) shows the area fraction of the two phases with *fcc* (bright contrast phase in BSED images) being 70% and *bcc* (dark contrast phase) being 30%. TEM examination from each of these regions revealed that while the *fcc* phase is disordered the *bcc* phase exhibits chemical ordering. The bright-field TEM image in Fig. 2(d) clearly shows the *fcc* phase to be the higher phase fraction phase. Convergent beam microdiffraction patterns from *fcc* and *bcc* phases are shown as insets in Fig. 2(d). The presence of extra super lattice spots at {001} positions in the [001]_*bcc*_ zone axis clearly indicates ordering within the *bcc* phase. An *fcc*/B2 microstructure have also been reported earlier in Fe_30_Ni_20_Mn_35_Al_15_ and AlCoCrFeNi_2.1_ EHEAs^10–18^, though the morphology of the eutectic microstructure varied with processing route and composition. Liao *et al*. showed that higher solidification rates not only influence the lamellar width but also the orientation relationship (OR) between the two phases^11,12^. There is a Kurdjumov–Sach (KS) OR between the *fcc*/*bcc* phases(highlighted in blue) wherein {111}*fcc*∥{110}*bcc and* 〈110〉*fcc*||〈111〉*bcc*. The pole figures shown in Fig. 2(c) also confirms the presence of KS OR between *fcc* and *bcc* phases. In general, multiple ORs including Kurdjumov–Sach (KS), Nishiyama–Wasserman (NW) and Pitsch are possible in *fcc*/B2 containing AlxCoCrFeNi alloys^22^.

The entropy of fusion of the two phases, comprising the eutectic, play a very important role in determining the morphology of the resulting microstructure. If both phases possess a low entropy of fusion, their growth is easy along all crystallographic directions and resultant microstructure is a regular lamellar eutectic. Where the low volume fraction phase in the eutectic possesses a high entropy of melting, as do intermetallic compounds, the eutectic microstructure is usually irregular^23^.

In the Al_0.7_CoCrFeNi alloy the B2 phase fraction is ~30% and shares an OR with the *fcc* phase. Consequently, this eutectic exhibits a lamellar-like morphology at a coarser scale, while at the microscopic scale the B2 lamellae are not continuous but are pinched off or broken in many regions, as revealed in Fig. 2(d). This can be probably because B2 is an intermetallic phase with high entropy of melting.

**Figure 3:**
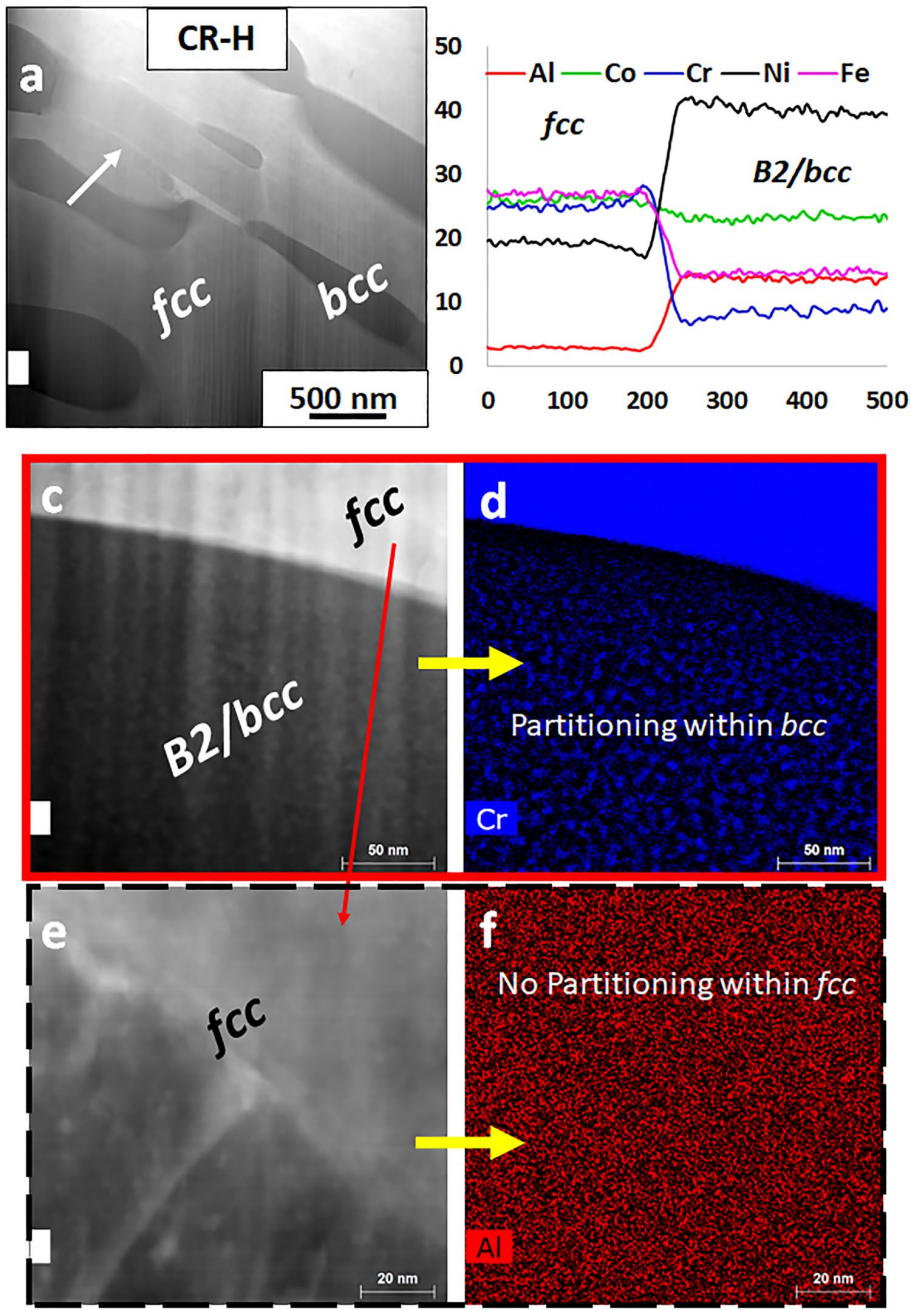
(**a**–**f**) STEM-EDS characterization of the Al_0.7_CoCrFeNi **CR-H** condition.

Figure 3(a) shows a HAADF-STEM image from the CR-H condition where the brighter phase is *fcc* and the darker phase is B2. One-dimensional compositional profiles for the various elements, across the *fcc*/B2 interface are shown in Fig. 3(b). These results are consistent with SEM-EDS results shown in Fig. 1. The compositions of the two phases are given in Supplementary Table T1. Higher magnification STEM-EDS maps from each phase are shown in Fig. 3(c–f). Note that compositional fluctuations are clearly visible within the B2 regions, as shown in the Cr map in Fig. 3(d) whereas the *fcc* region does not exhibit such fluctuations (refer to Fig. 3(f)). These fluctuations suggest the presence of a two-phase mixture of possibly B2 (ordered) containing Cr rich nano-precipitates of a disordered *bcc* phase. This is consistent with the observed diffraction pattern shown in Fig. 2(c) since the overlap of B2 + *bcc* diffraction patterns along the same zone axis cannot be distinguished from that of a single B2 phase along the same zone axis. Based on the STEM-EDS results and the diffraction pattern, it can be concluded that the B2 lamellae in this EHEA are actually two-phase mixtures of B2 + *bcc*. Such a decomposition of the B2 phase into B2 + *bcc* has been previously reported in Al-Co-Cr-Fe-Ni HEAs by Gwalani *et al*.^3^.

**Figure 4:**
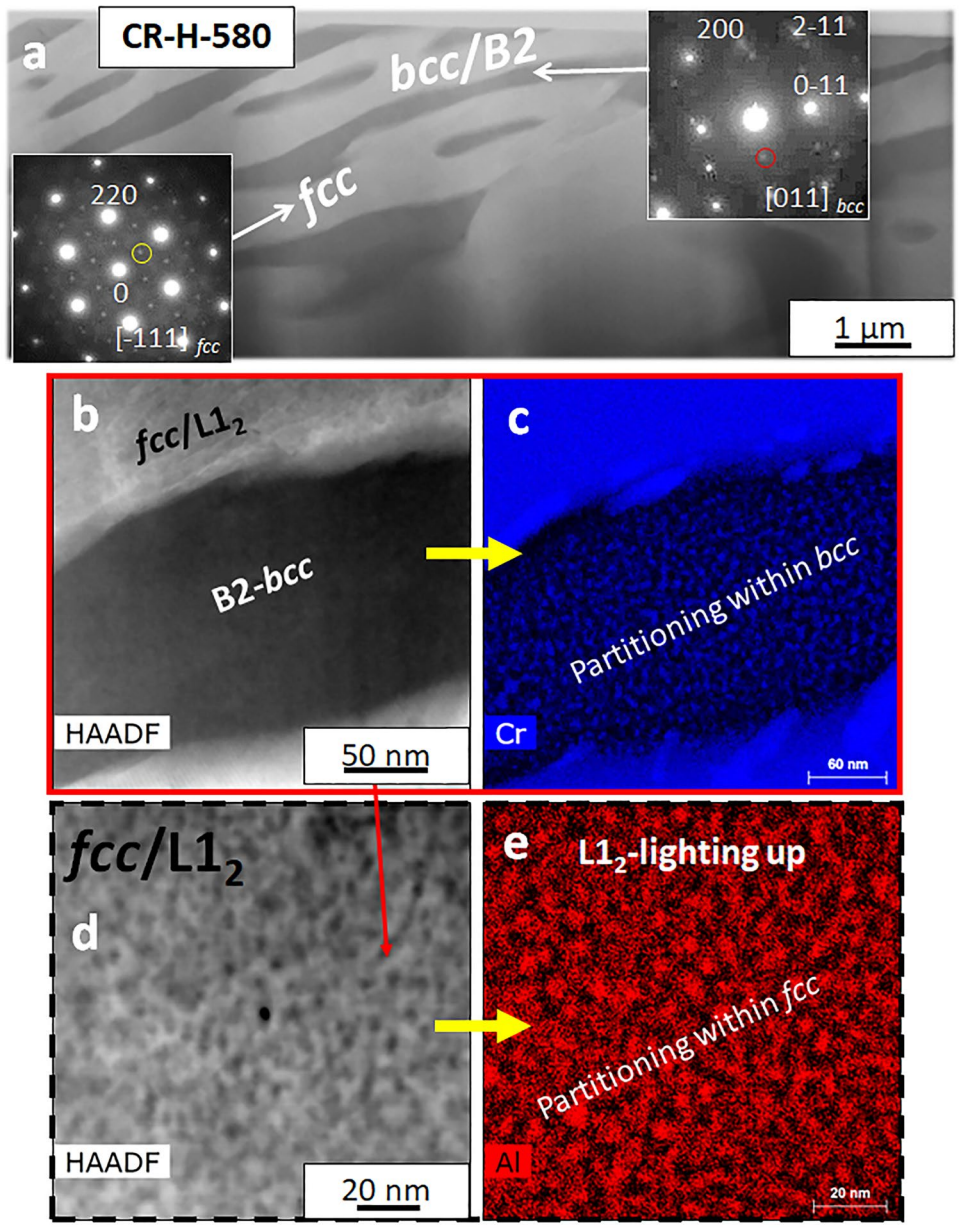
(**a**–**e**) STEM-EDS characterization of the Al_0.7_CoCrFeNi **CR-H-580** condition.

In the CR-H-580 sample, the microstructure is similar to CR-H condition and is shown in Supplementary Fig. S1. Figure S1(a) shows the BSED image with lamellae of *fcc* and B2/*bcc* phases. This is clearly revealed by the EBSD results shown in Fig. S1(b,c). A STEM image from this condition is shown in Fig. 4(a) and the SAD patterns from the two phases are shown as insets. The *bcc*/B2 region is similar to the CR-H condition where Cr rich *bcc* nanoprecipitates are present within the B2 phase (Fig. 4(b,c)). The superlattice spots at {001} positions can be noted in the [011]_*fcc*_ zone which are not seen in CR-H condition. This clearly shows the ordering of the *fcc* phase as well in CR-H-580 condition. A compositional partitioning within *fcc* as shown in Fig. 4(d,e) further confirms the presence of *fcc* + L1_2_ (ordered *fcc*) phase in this condition.

**Figure 5:**
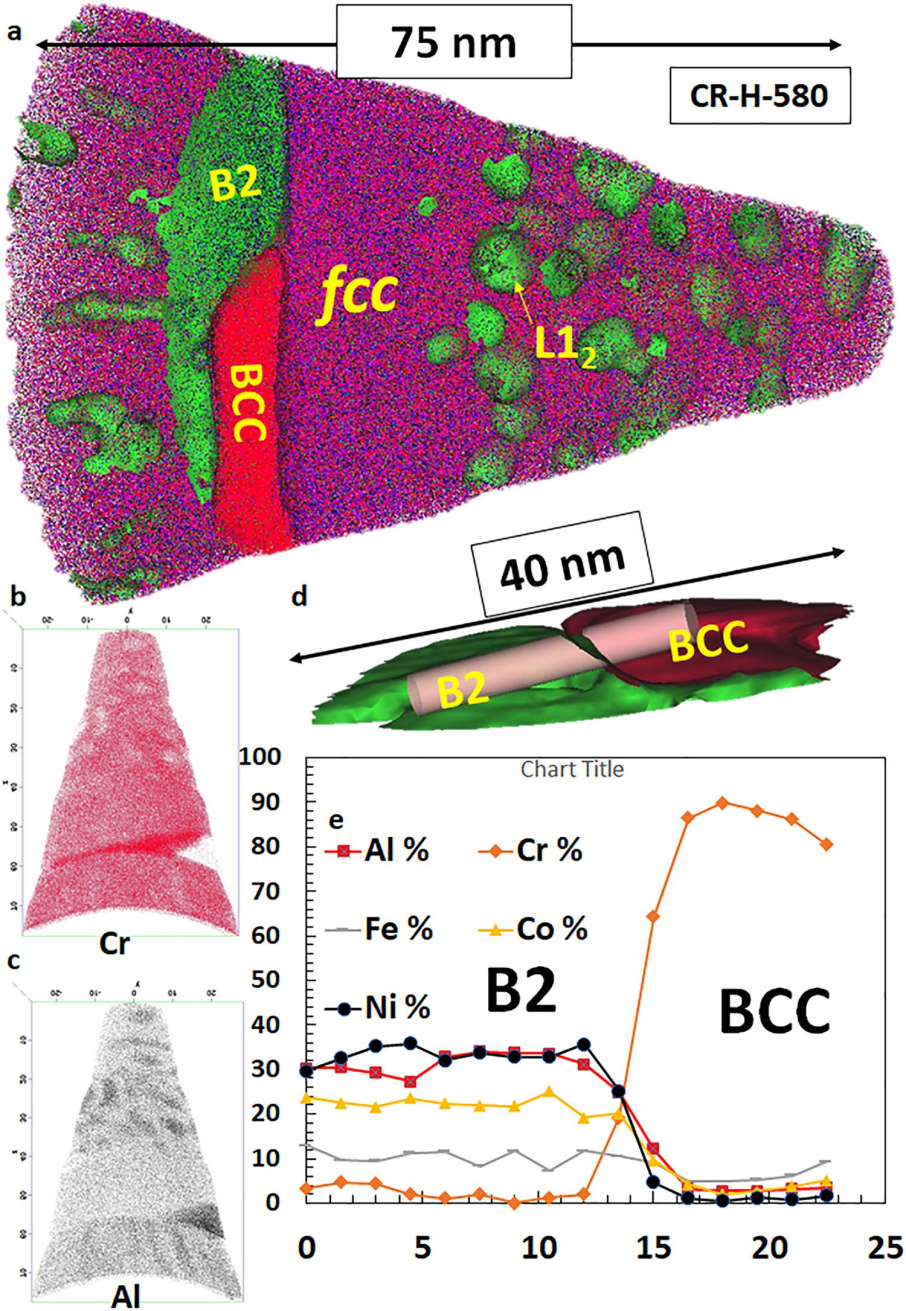
APT results from CR-H-580 condition (*fcc*/*bcc* interface).

Atom probe tomography results from the *CR-H-580* condition are shown in Supplementary Fig. S2 and Fig. 5. Supplementary Fig. S2 shows a reconstruction from an *fcc* + L1_2_ region of the microstructure. Fine-scale spherical precipitates with a radius of ~3–5 nm are uniformly distributed within the *fcc* lamellae. The compositional partitioning across the matrix-precipitate interface is shown by a proximity histogram in the figure. The L1_2_ precipitates are rich in Ni (~55 at. %) and Al (~35 at. %) whereas the matrix consists mainly of Cr (~29 at. %), Fe (~26 at. %) and Co (~25 at.%) with smaller amounts of Ni (~13 at.%) and Al (~7 at.%). The volume fraction of the precipitates is ~9.5% of the total volume of the reconstruction. The compositional details of the B2 and *bcc* are revealed in another reconstruction shown in Fig. 5. Figure 5(a) shows the *fcc*/L1_2_ region near the tip of the reconstruction with a sliver of B2/*bcc* region has been captured in the lower portion. The *bcc* is identified by the enrichment of Cr (Fig. 5(b)) and B2 contains high concentration of Ni and Al (Al enrichment can be seen in Fig. 5(c)). Note that the Ni concentration is lower in the B2 as compared to the L1_2_ (~55 at. % in L1_2_ and ~30 at. % in B2). The iso-concentration surfaces of Al (green) and Cr (red) are used to delineate the B2/*bcc* phases in Fig. 5(d) and a cylinder is used to map the 1-dimensional compositional profile (shown in Fig. 5(e) across the two phases. The exact compositions of each phase measured from the APT analysis (also provided in Supplementary Table T2) are as follows (all in at. %):

***Fcc***: ***Al***-*6.7,* ***Co****-24.1,* ***Cr****-28.8* ***Ni****-14.1 and* ***Fe****-26.3* *(Al*_*0.25*_*CoCrFeNi*_*0.5*_*)*

***L1***_***2***_: ***Al****-35.8,* ***Co****-4.3,* ***Cr****-0.9,* ***Ni****-56.4, and* ***Fe****-2.6 (Ni*_*2.26*_*, Co*_*0.2*_*, Fe*_*0.1*_*, Cr*_*0.04*_*, Al*_*0.4*_*) Al*

***B2***: ***Al****-33.7,* ***Co****-21.7,* ***Cr****-0.1,* ***Ni****-32.8, and* ***Fe****-11.7 (AlCo0.7NiFe0.4)*

***Bcc***: ***Al****-1.9,* ***Co****-2.7,* ***Cr****-90,* ***Ni****-0.5, and* ***Fe****-4.9*

Note that the ordered L1_2_ phase in this alloy contains an unusually high amount of Al. The substantially higher 35.8 at. % Al in this L1_2_ phase, as compared to the stoichiometric 25 at % based on Ni_3_Al, suggests that Al anti-site defects are created on the Ni sites of the L1_2_ phase in this alloy. However, detailed first-principles, electronic structure based computational studies need to be coupled with these experimental observations in order to establish the site-occupancies of different elements within the ordered L1_2_ structure. Interestingly, a similar L1_2_ composition was reported in case of the Al_0.3_CoCrFeNi HEA as well^3^.

In case of the B2 composition (which is based on a NiAl structure), if we assume that Fe occupies the Ni sites then the approximate formula for the B2 phase (with anti-site defects) could be –(NiFe)(AlCoCr).

#### Effect of microstructure on mechanical properties

**Figure 6:**
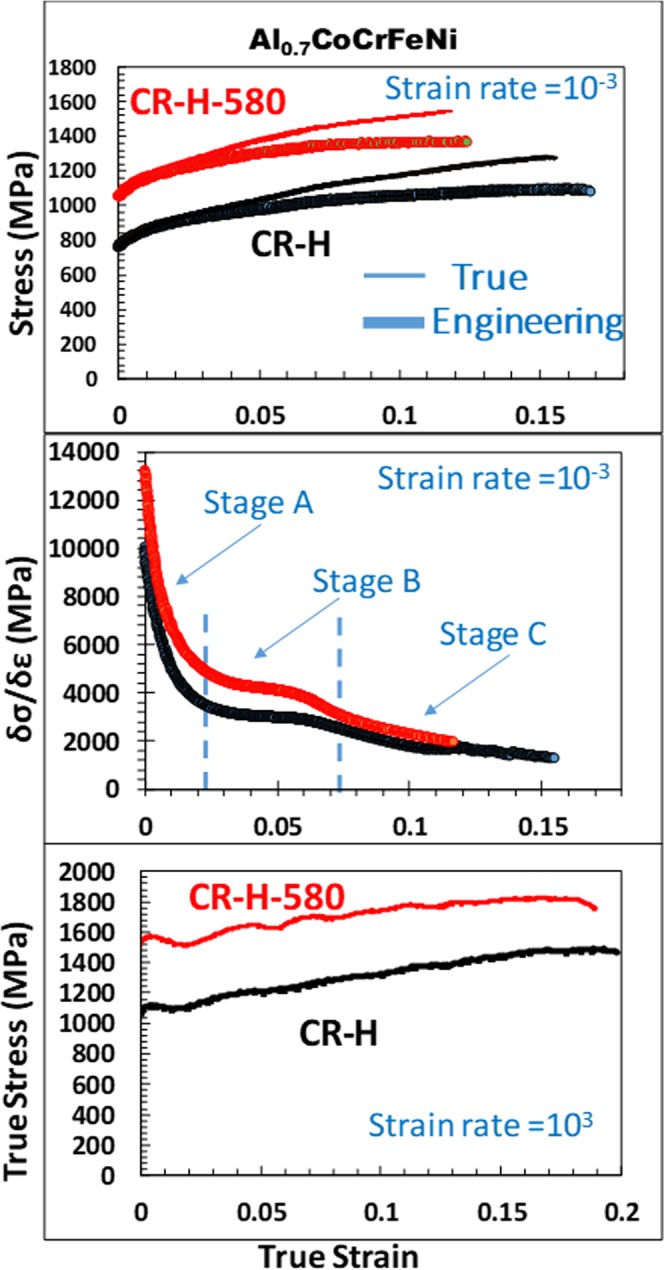
True stress-strain plots from CR-H (aged at 1100 °C for 5 min) and CR-H-580 (aged at 1100 ^o^C for 5 min followed by aging at 580 °C for 24 h) at two different strain rates as labeled.

The advantage of such a two-phase eutectic alloy is that it consists of a more easily deformable ductile (*fcc* or *fcc* + L1_2_) phase, and a hard, difficult to deform, B2 + *bcc* phase. The hard B2 + *bcc* phase defines the inter-lamellar spacing of the softer *fcc* phase. To evaluate the mechanical performance of the Al_0.7_CoCrFeNi alloy, the CR-H and CR-H-580 conditions were tested under two different strain rates (10^−3^ s^−1^ and 2 × 10^3^ s^−1^) and the corresponding stress-strain plots are shown in Fig. 6. Figure 6(a) shows the engineering (thick line) and true (thin line) stress-strain plots from quasi-static testing. The CR-H condition with *fcc* + B2 (with fine scale *bcc* precipitates) shows a yield strength (YS) of ~770 MPa, UTS ~1090 MPa with an elongation to failure (EF) of ~17% under the quasi-static deformation (10^−3^). The CR-H-580 condition with the *fcc* + L1_2_ & B2 + *bcc* microstructure exhibited a YS ~1080 MPa, UTS ~1370 MPa and EF of ~15%. A precipitation strengthening of ~310 MPa is observed after the additional heat treatment with just ~2% reduction in the elongation.

The strength enhancement due to nano-precipitates can be estimated by using a simple Orowan strengthening model based on the dislocation bowing and by-pass mechanism. It can be described by the equation below:

1 $${\rm{\sigma }}\text{Orowan}=\frac{{\bf{G}}\ast {\bf{b}}}{{\boldsymbol{\lambda }}}$$

2 $${\rm{\lambda }}=\frac{{\bf{4}}({\bf{1}}-{\bf{f}}){\bf{r}}}{3{\bf{f}}}$$

Where λ is average planar center-to-center distance between nano-precipitates, G is the shear modulus of the matrix, b is the Burgers vector (assumed to be 80 GPa and 0.255 nm for CoCrFeNiMn respectively^4^, r is the radius of the nano-precipitates (3 nm), and f is the volume fraction of precipitates, which is 9.5% by vol. in fcc phase (from APT reconstruction) and is equal to $9.5\ast 0.7=6.65 \%$ by vol. in the entire microstructure. Based on Eqs (1) and (2), the Orowan strengthening can be estimated to be ~360 MPa, which compares reasonably well with the experimentally observed strengthening value of 310 MPa.

This alloy strain-hardens substantially under both heat treatment conditions (refer true stress-strain plots in Fig. 6(b)). The work-hardening rate (δσ/δε) versus true strain is shown in Fig. 6(b). Both the heat treatment conditions depict different stages of strain hardening commonly observed in a low SFE material^24–26^ (labeled in Fig. 6(b)). In Stage A, strain hardening shows the steady decrease in the hardening rate which signifies the dislocation mediated plasticity, stage B has a near constant hardening rate and in stage C the hardening rate again decreases steadily. The initiation of stage B has been correlated with the initiation of deformation twinning. Similarly, stage C is correlated with extensive twin intersections in the microstructure^24^. The decrease of strain hardening in stage C is due to the decreasing rate of primary twinning. In the current study, the steady-state work-hardening rate is marked as stage B in the work-hardening rate versus true strain, in the Fig. 6(b). The steady state work hardening for CR-H condition is ~3000 MPa whereas for CR-H-580 is >4000 MPa. Hence, the precipitation of ordered L1_2_ nano-precipitates strengthened the alloy and improved the strain hardening rate. The details of the associated deformation mechanisms will be discussed further in later section.

A SHPB apparatus was used to conduct the high strain rate (dynamic) deformation test at a strain-rate of 2 × 10^3^ s^−1^. None of the specimen fail till 20% straining at 2 × 10^3^ s^−1^ strain-rate. We note that the flow stresses are evidently greater at the high strain rate testing. Hence a strong strain-rate sensitivity (SRS), which is a measure of strengthening of a material at higher strain rates, is observed. For the force equilibrium within the specimen, the elastic waves generated in the SHPB technique needs at least an eight time reverberation^27^. The stress estimation measured from elastic wave pulses are reliable only after this time. Therefore, as a common practice the stress values prior to 1% strain in the curve are disregarded. The flow-stress in the CR-H condition (at ~1% true strain) is ~1080 MPa and that for CR-H-580 is ~1440 MPa.

The parameter for strain rate sensitivity (SRS), m, is calculated based on the flow stresses at two different strain-rates as following:

3 $$m={[\frac{\partial \mathrm{ln}\sigma }{\partial \mathrm{ln}\dot{\varepsilon }}]}_{T,\varepsilon }$$

σ is the flow stress, ${{\rm{\varepsilon }}}^{\cdot }$ is the strain-rate, T is temperature, and ε denotes strain. The strain rate sensitivity parameter, m at 1% flow stress for the two alloy conditions can be calculated to be 0.017 for CR-H-580 and 0.018 for CR-H condition. A recent study by Li *et al*.^28^ on single phase *fcc* Al_0.3_CoCrFeNi HEA showed a very high SRS of 0.053. The lamellar dual phase microstructure has a high density of interphases, which increase strength significantly compared to a single phase large grained high entropy alloy^28^, however they also lessen SRS considerably. The strain rate sensitivity is not affected as much by precipitation of coherent L1_2_ precipitates.

Gangireddy *et al*.^29^ examined the microstructural dependency of SRS and concluded that relative contributions from short-range (thermal) and long-range (athermal) obstacles to dislocation motion, determine strain rate sensitivity of yield strength. As slip is the preceding mechanism for deformation, before other mechanisms such as twinning or transformation-induced plasticity can initiate, at small plastic strains, before the differences in deformation mechanisms can affect strength, the yield strength is determined by obstacles to slip alone. The amplitude of long-range obstacles is too large for thermal activation and therefore can be treated as athermal in nature and have no/little dependence on strain rate/temperature. While the short-range obstacles, where thermal activation can help to overcome such barriers, brings the strain rate dependency. Even though fine-scale L1_2_ precipitates increased strength by 30% in aged CR- H-580, they are coherent in nature and thermally activated. So, SRS drops only slightly in this microstructure.

Within the *fcc* + L1_2_ microstructure of the *fcc*-based lamellae shown in the APT reconstruction in Fig. 5, there appears a precipitate free channel near the interface of the B2 + *bcc* and *fcc* + L1_2_ lamellae (refer Fig. 5). Such precipitate-free channels near the lamellar boundaries were also observed in the high-resolution STEM-EDS maps. These precipitate-free channels can be relatively softer as compared to the fcc + L1_2_ regions, and consequently act as sites for high dislocation storage in the vicinity of the interfaces during deformation. Such a mechanism could potentially rationalize the similar strain rate sensitivity observed in case of the Al_0.7_CoCrFeNi eutectic HEA, in the absence or in the presence of L1_2_ precipitates within the fcc-based lamellae.

#### Post Deformation characterization

**Figure 7:**
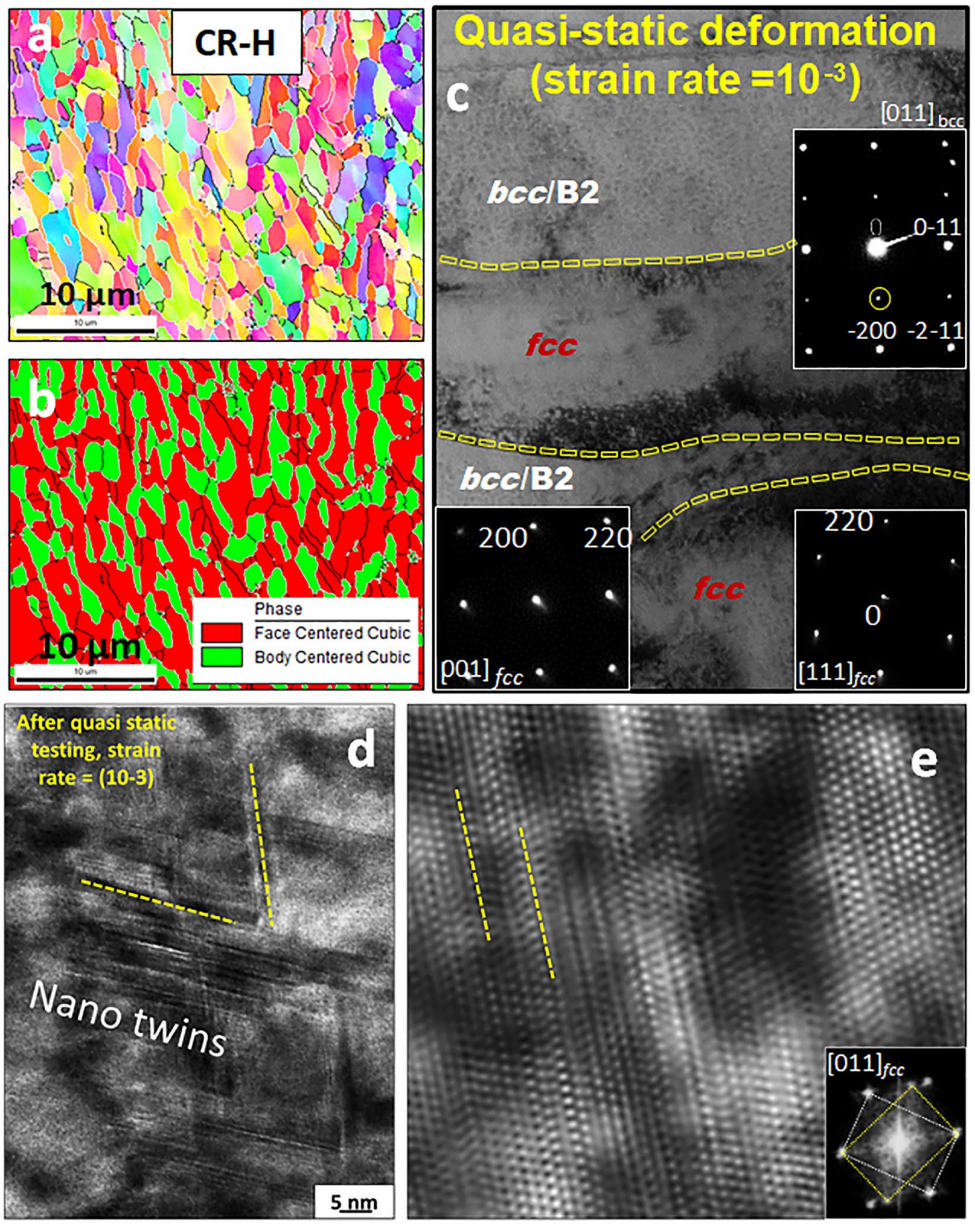
EBSD and TEM results from CR-H condition of Al_0.7_CoCrFeNi HEA after quasi-static testing.

Figure 7 shows the EBSD and TEM results from the CR-H condition after quasi-static deformation. Figure 7(a,b) show the IPF and phase map, where a clear difference in the *fcc* and *bcc* regions can be observed, when compared to before deformation structure. The *bcc* phase is broken into smaller grains and segmented. It is difficult of notice any deformation twin at this magnification. More details are obtained upon TEM examination. A TEM foil was FIB prepared from a region near to the failure under quasi-static tensile testing. A BFTEM image showing the *fcc* and *bcc* regions is displayed in Fig. 7(c). The SADPs from [001] _*fcc*_ and [111]_*fcc*_ are shown in the bottom of the image whereas the SADP from the [011]_*bcc*_ is shown on the top right of the image as the inset. The presence of the super-lattice spots in the [011]_*bcc*_ (highlighted by yellow circle in the inset image) clearly establishes that the chemical ordering in this region is still present after deformation. The *bcc*/B2 phase in Al_X_CoCrFeNi has been reported to be much stronger compared to the *fcc* phase^1^. Liao *et al*. used transmission electron microscopy (TEM) on an *fcc*/B2 eutectic alloy, that the plastic deformation essentially took place in the softer *fcc* phase with nanohardness of 2.7 GPa and few dislocations were seen in the harder B2 phase with nanohardness of 4.4 GPa^11^. The *fcc* phase deforms first resulting into strain mismatches at interfaces and heterogeneous deformation-fields. That can in turn result in accumulation internal stresses accommodated by larger storage of geometrically-necessary-dislocations (GND) at these interfaces^28^. The interfaces can act as the barriers to dislocation-motion^30^. The post-test microstructures in Fig. 7(c) shows a heavy pile-up of dislocations in the softer *fcc* phase at the *fcc*-B2 interface. The fine-scale lamellae sizes would elevate twinning stress and retard twinning^25^. Due to high twinning stresses, phenomena like dislocation locking have been reported to be dominant^30^. These barriers can increase with tendency for slip planarity due to relatively low SFE, high Peierls-Nabarro stresses, and ordered phases (in the case of CR-H-580).

There is no clear evidence of large-scale twinning in any of the two regions. However, on examination at a higher magnification in HRTEM mode, fine scale faults/nano-twins are observed as shown in Fig. 7(d,e). Figure 7(d) shows the HRTEM of nano-twins along [011]*fcc* in the CR-H condition after quasi-static testing to failure. Fourier filtered image in Fig. 7(e) depicts the atomic resolution of the fine-scale, few atomic planes thick twins. The corresponding Fourier filter transform (FFT) is shown as an inset. This condition shows high density of nano-twins but no thicker twins are observed. The elemental composition of *fcc* phase is comparable to ~Al_0.1_CoCrFeNi (refer Supplementary Table T1). Choudhuri *et al*. investigated Al_0.1_CoCrFeNi alloy and reported nano-scale twinning in the alloy after quasi-static deformation, the deformation behavior of the *fcc* phase in the current alloy can be compared to that^31^. The high work hardening behavior of the current alloy can be due to the high density of nano-twins and large dislocation accommodation at the long range obstacles in form of *fcc*-B2 interfaces.

**Figure 8:**
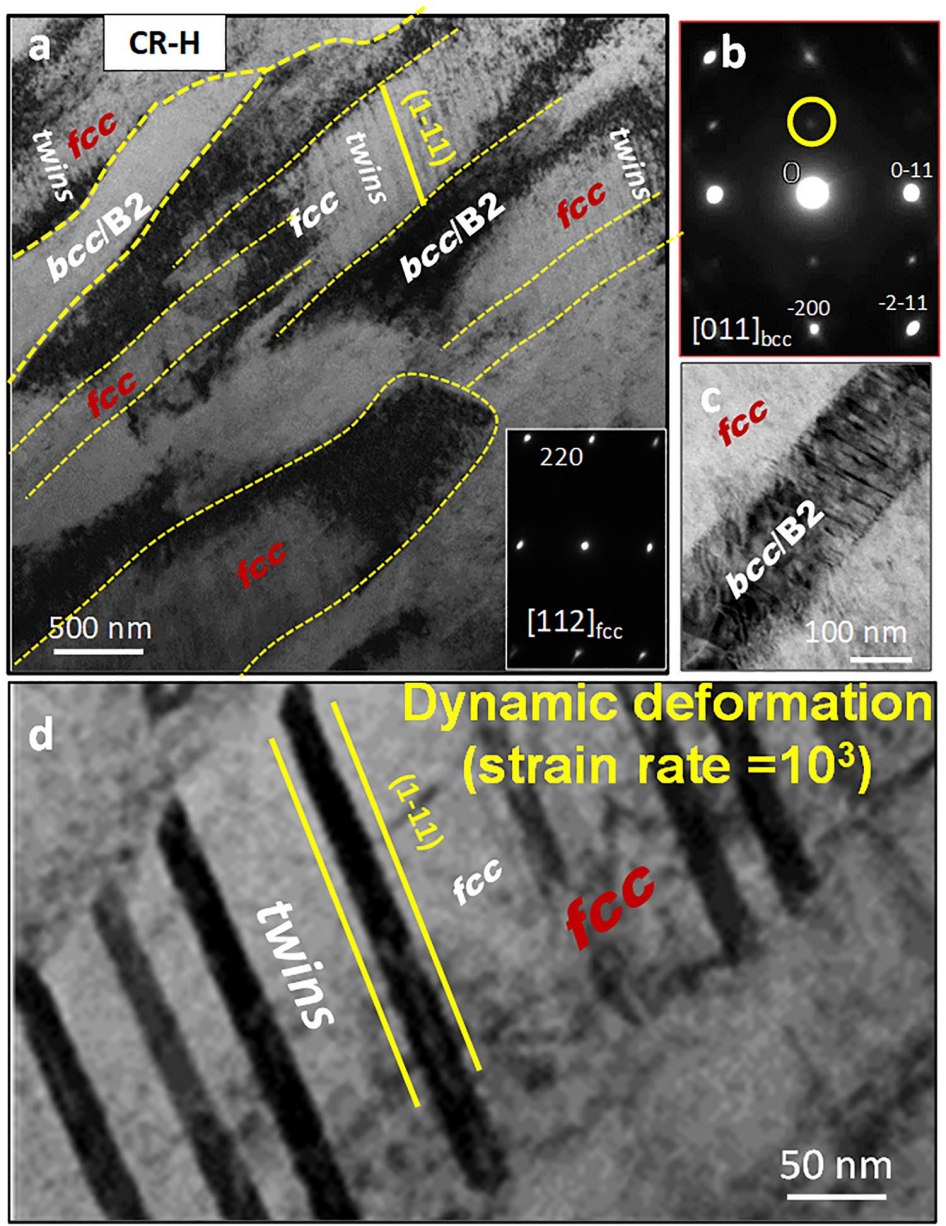
TEM results from CR-H condition of Al_0.7_CoCrFeNi HEA after dynamic testing.

Now we present the TEM results from the dynamically deformed (strain rate = 2 × 10^3^) CR-H sample. Figure 8(a) shows a low magnification BFTEM image showing the *fcc* and *bcc* regions as labeled in the figure. A stark difference here on comparison to the quasi-static condition is the high density of twins in the *fcc* region. The SADP taken from the *fcc* region is shown as the inset to the Fig. 8(a) and that from the *bcc*/B2 region is shown in Fig. 8(b). The *bcc*/B2 region has highly faulted structure as can be seen in Fig. 8(c). A high magnification image showing the twins in the *fcc* phase is shown in Fig. 8(d). The twin thickness is about 20 nm which is much thicker compared to deformation under quasi-static testing.

Investigation on CR-H-580 sample after deformation is shown in Supplementary Fig. S3. Figure S3(a,b) show results from the quasi-static testing and Fig. S3(c) shows the results from the dynamically tested sample. Note, the super-lattice spots, in the ZA = [011]_*fcc*_ SADP shown in the inset of Fig. S3(a) after quasi static testing and in ZA = [112]_*fcc*_ SADP shown as the bottom inset of Fig. S3(c) after dynamic testing, are absent. Hence, the L1_2_ phase disorders after deformation under both strain rates. Dissolution of L1_2_ on deformation has been discussed in literature both in superalloys as well as in HEAs^16,32^. The L1_2_ disorders when the dislocations shear through them and form anti-phase boundaries. The anti-phase boundaries can further increase the strain hardening rate of the alloy. An increase in the strain hardening rate (refer Fig. 6(b)) can be explained by disordering of L1_2_ precipitates and formation of anti-phase boundaries. Wani *et al*.^16^ suggested that microbands and shear bands could be involved in the disordering process.

Comparing the propensity of twinning under the two different deformation rates, the twinning increases sharply under high strain rate testing as seen in Fig. S3(c). The B2 region remains ordered as seen in the ZA = [011]_*bcc*_ SADP which shows presence of the super-lattice spots in the top inset of the Fig. S3(c). Deformation in the *fcc* phase can be explained by collective dislocation glide and nucleation governed plasticity while that of the B2 phase is due to dislocation cross-slip. This is further investigated by TEM analysis to show the twinning elements in *fcc* at high strain rate.

## Conclusions and Summary

The effect of thermo-mechanical treatment on phase evolution and mechanical properties in an *fcc*-based Al_0.7_CoCrFeNi HEA was investigated. While the dual phase eutectic Al_0.7_CoCrFeNi was precipitation strengthenable by introducing hard-coherent intermetallic phase in the *fcc* phase via suitable heat treatment, post deformation TEM examination was used to investigate the modes of deformation under various strain rates. The following can be established from the current study:

1. *Phase composition*
  The alloy was cast, homogenized, rolled and heat treated at two different temperatures i.e. 1100 °C and 580 °C. It was observed that the even on water quenching (fast cooling) from 1100 °C(CR-H condition), the B2 to B2 + *bcc* transformation in the B2 lamellae takes place. However, *fcc* phase can be retained as disordered in CR-H condition. Another heat treatment was conducted on the CR-H condition by annealing the alloy at 580 °C for 24 h. This heat treatment introduced nano-scale L1_2_ precipitates in the *fcc* lamellae. APT results revealed the compositions of the *fcc*, L1_2_, B2 and *bcc* phases.
2. *Mechanical testing*
  The alloy was tested under quasi-static (strain rate = 10^−3^) tensile loading and dynamic (strain rate = 2 × 10^3^) compressive loading. Introduction of a large density of coherent nano-scale L1_2_ precipitates in the *fcc* phase resulted in significant YS increase in the alloy. The *fcc* & L1_2_ + B2 & *bcc* microstructure exhibited an yield strength of ~1000 MPa, ultimate tensile strength (UTS) of ~1400 MPa and elongation to failure of ~13% under quasi-static loading whereas flow stresses ~1800 MPa under dynamic loading. The strengthening contribution due to fine scale L1_2_ is modeled using Orowan dislocation bowing and by-pass mechanism.
  L1_2_ precipitates disorders on deformation under both quasi-static and high strain rate. Post deformation transmission electron microscopy analysis revealed that twinning is observed in *fcc* phase under high strain rate deformation, which was minimal under quasi-static loading.
3. *Work hardening rate*
  Drawing from the work hardening plot (Fig. 6(b), the alloy is expected to show immense propensity to deformation twinning, but after post deformation examination such is not observed. Despite of low deformation twinning seen after quasi-static testing, the high work hardening in the CR-H condition could be due to higher dislocation storage capacity at the interfaces and dislocation locking mechanism. In CR-H-580 under quasi-static testing the disordering of L1_2_ phase resulting in formation of anti-phase domains can further increase the strain hardening in the alloy.
4. *Strain rate sensitivity (SRS)*

Even though fine-scale L12 precipitates increased strength by 30% in aged CR-H-580, they are coherent in nature and thermally activated. So SRS drops only slightly in this microstructure. The precipitate free channel near the interface of the B2 + bcc and fcc + L12 lamellae (refer Fig. 5) can be relatively softer as compared to the fcc + L12 regions, and consequently act as sites for high dislocation storage in the vicinity of the interfaces during deformation. Such a mechanism could also potentially rationalize the similar strain rate sensitivity observed in case of the A_l0.7_CoCrFeNi eutectic HEA, in the absence or in the presence of L12 precipitates within the fcc-based lamellae.

## Supplementary information

Supplementary Figures and Tables

## Data Availability

All the background data and information is available with the corresponding author and can be presented on a reasonable request.
