# Supplementary material for: Influence of ordered L12 precipitation on strain-rate dependent mechanical behavior in a eutectic high entropy alloy
Source: Sci Rep. 2019 Apr 23;9:6371. doi: 10.1038/s41598-019-42870-y (PMC6478865; doi:10.1038/s41598-019-42870-y)
Supplement: Supplementary file 1 — Supplementary Figures and Tables [file 41598_2019_42870_MOESM1_ESM.docx]

**Influence of ordered L1_2_ precipitation on strain-rate dependent mechanical behavior in a eutectic high entropy alloy**

Bharat Gwalani^a,b#^, Sindhura Gangireddy^a^, Yufeng Zheng^c^, Vishal Soni^b^, Rajiv S. Mishra^a,b^, Rajarshi Banerjee^a,b*^

^a^Advanced Materials and Manufacturing Processes Institute, University of North Texas, Denton, TX 76207

^b^Materials Science and Engineering, University of North Texas, Denton, TX 76207

^d^Department of Materials Science and Engineering, The Ohio State University, Columbus, OH 4310, USA

^#^ Now at Physical and Computational Sciences Directorate, Pacific Northwest National Laboratory, USA

**Corresponding author: raj.banerjee@unt.edu*

**Supplementary Information
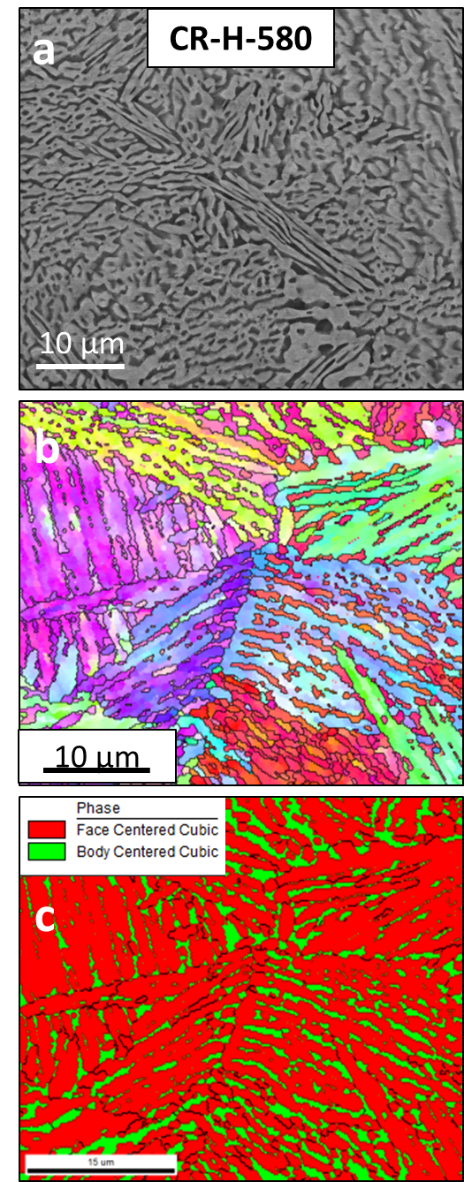
**

**Figure S1 (a-c)** SEM-EBSD characterization of the Al_0.7_CoCrFeNi CR-H-580 condition.

**
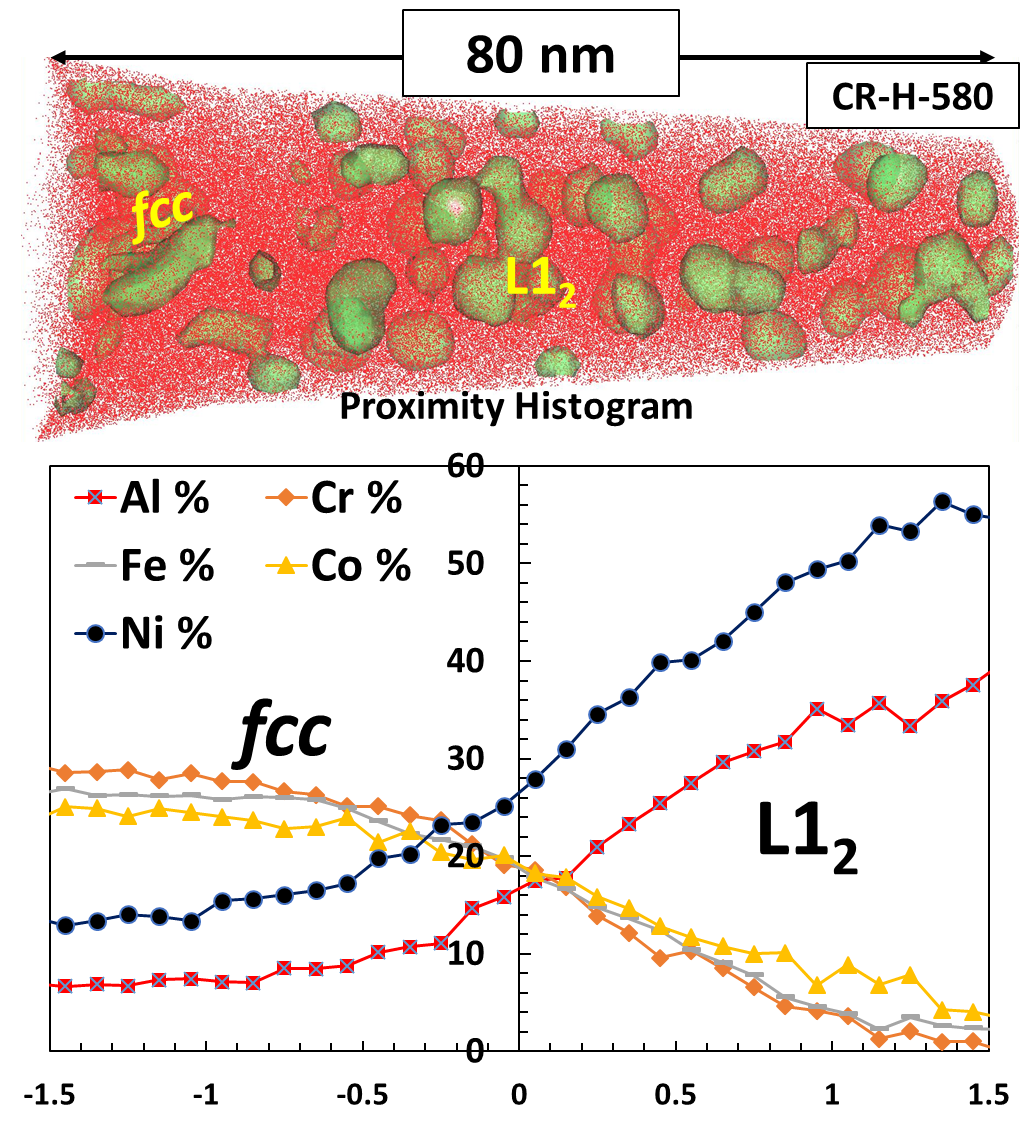
Figure S2** APT results from CR-H-580 condition (*fcc*/L1_2_ region)

**
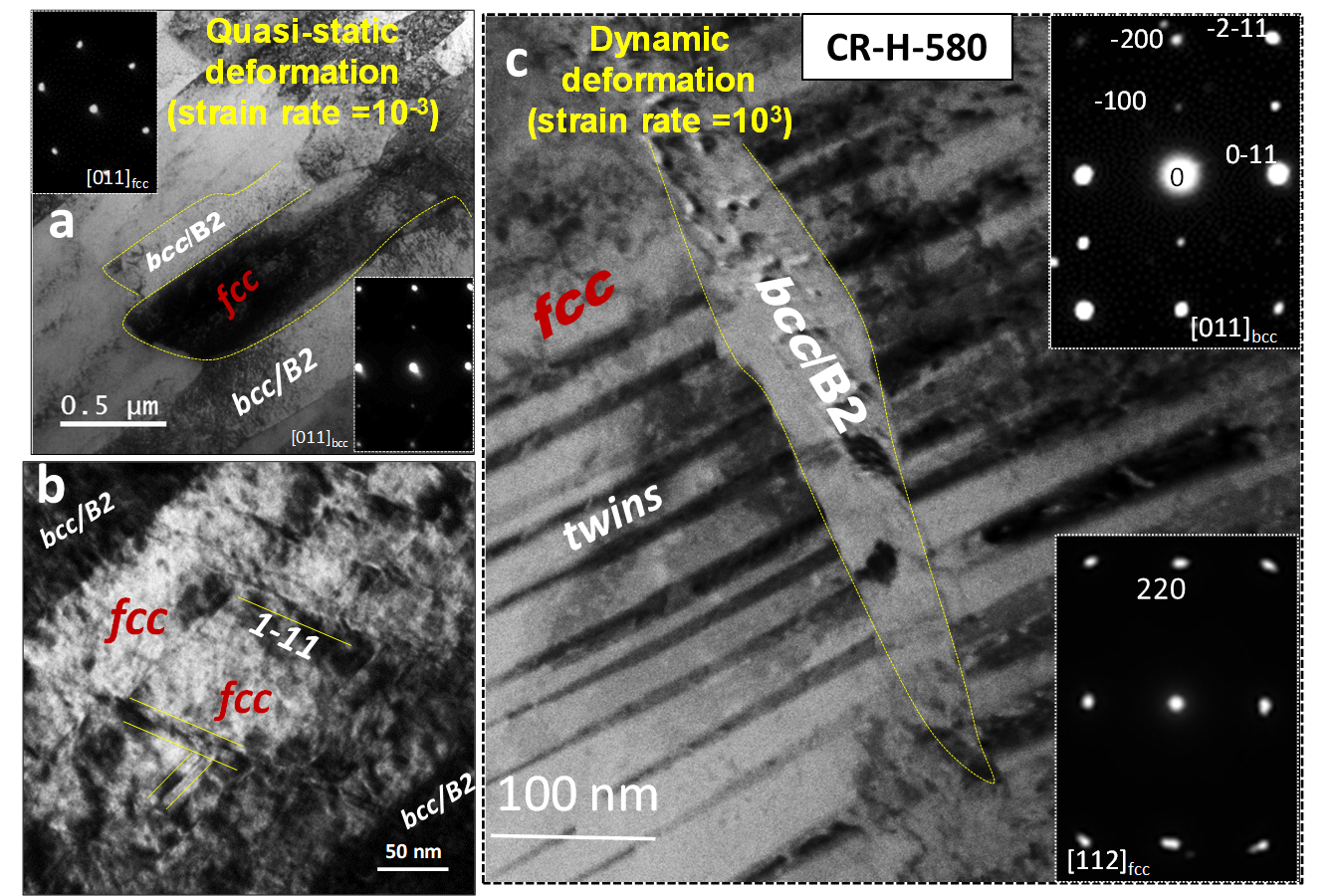
Figure S3** TEM results from CR-H-580 condition of Al_0.7_CoCrFeNi HEA after quasi-static and dynamic testing.

**Table T1 Chemical composition obtained from STEM-EDS**

**
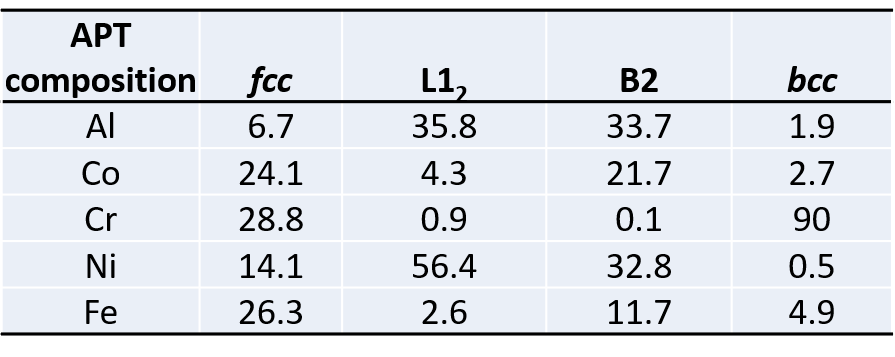

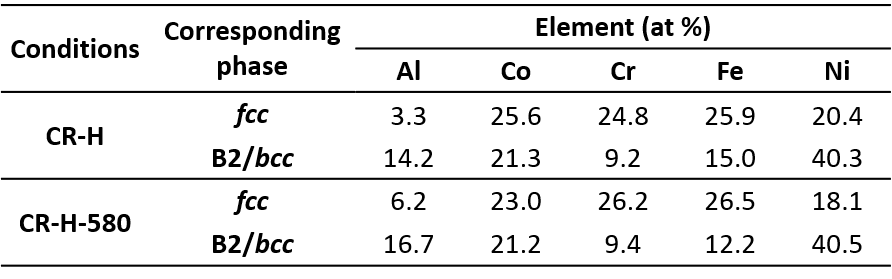
**

**Table T2 Chemical composition obtained from APT**
